# Dr Val Vallyathan: in memoriam

**DOI:** 10.1186/1743-8977-7-24

**Published:** 2010-09-10

**Authors:** Vincent Castranova, Jeff Fedan

**Affiliations:** 1 National Institute for Occupational Safety and Health, 1095 Willowdale Road Morgantown, WV 26505

## 

Val Vallyathan died in a tragic accident Friday, July 23, 2010 while visiting family in New Jersey. His colleagues at NIOSH, Morgantown, WV are stunned by his sudden death.

Val was married for 45 years to Usha, and they were blessed with two children, Sanjay and Veena, and two grandchildren.

Val received his B.Sc. (Honors), M.Sc., and Ph.D. degrees at Maharaja Sayajirao (M.S.) University of Baroda, India. He next held several academic and research and teaching positions at Maharaja Sayajirao University, the University of Baroda, the University of Guelph (Canada), the University of Vermont, and the Institute for Muscle Disease. Val joined NIOSH in 1979 and had been a leading researcher on the Morgantown campus ever since. He served as an Advisor to many university graduate students and postdoctoral fellows at the Maharaja Sayajirao University, the University of Vermont, West Virginia University and NIOSH.

At NIOSH Val was a Research Physiologist and Team Leader in the Pathology and Physiology Research Branch (PPRB) of the Health Effects Laboratory Division. In fact, Val was trained primarily as an experimental pathologist, and his primary research interests lay in occupational respiratory diseases. After his retirement in October, 2009, Val came back to PPRB as an Expert Consultant; he was unable to cut his ties to his research activities, and probably would have never really retired and left his research.

In addition to his duties at NIOSH, Val held several Adjunct Professorships at West Virginia University, Morgantown, WV, in the Departments of Pathology, Basic Pharmaceutical Sciences, and Anatomy.

Val's influence on occupational health research was widely acknowledged and reached around the world. Of the many conferences he organized or played a significant role in, he would be most proud of the four international conferences on oxygen and nitrogen free radicals, the last one being held in October, 2009. Other conferences with which he was involved, both within and outside of NIOSH, were equally relevant, timely and successful.

Nominated 17 times for Alice Hamilton and Charles C. Shepard Awards for "Best Paper of the Year" in NIOSH or CDC, respectively. Val received the Hamilton award in 1999 and 2001 and honorable mentions in other years.

**Figure 1:**
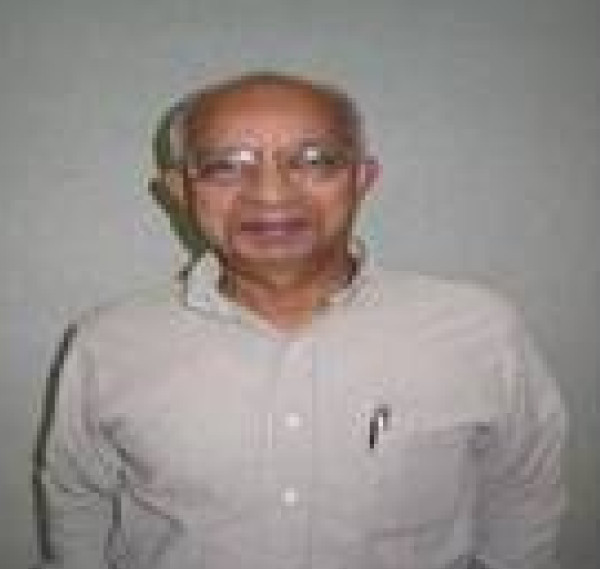
**Dr Val Vallyathan**.

Val (figure 1) was internationally recognized in his field, and served as external reviewer and expert consultant for many federal research agencies, universities and organizations. He gave scores of invited presentations around the world at conferences, universities and workshops. He enjoyed being of service to others and gave freely of his knowledge; he was repeatedly turned to for scientific advice.

In addition to holding one patent, Val published extensively. He served as a book editor, guest editor or chapter or review contributor for many volumes. In addition, he authored at least 376 full publications in scientific journals, with another dozen or so invited articles. He has published at least 198 conference presentations and abstracts, with 5 papers in preparation at the time of his death. What we noticed as we reflected on Val's tenure at NIOSH was the remarkable diversity of his interests and curiosity. For example, he has published papers characterizing the effects, mechanisms of action, and possible approaches for preventing disease from the following array of toxic agents: nanotubes, metal particles, asbestos, silica, quartz, cigarette smoke, agricultural dust, coal dust, reactive oxygen species, grape dust, citrus dust, diesel exhaust, cadmium, iron, mercury, cumene hydroperoxide, toluene diisocyanate, welding fume, arsenic, chlorogenic acid, lipopolysaccharide, vanadate, chromium, nickel, eugenol, nylon flock, volcanic ash, and talc. His work also paid much attention to pathways involved in apoptosis, the respiratory burst and surfactant's role in the lungs. Of these subjects his greatest scientific contributions were to the understanding of silicosis and the roles of free radicals in lung disease.

The above facts are a dry, statistical summary of the scientific life of Val Vallyathan. They do nothing to honor him as a person, friend and colleague. Val was universally liked by everyone; he had malice for no one. It was always enjoyable to be in Val's company because he was respectful, engaging and pleasant. He was open, honest and unpretentious, despite his accomplishments. Rarely did he ever complain, but, if he did, he did so with elan, wit and wisdom, making us all laugh and ever aware of the special person he was.

We have lost a dedicated and committed lung researcher and friend. His is a huge legacy.

